# A Zebrafish Model of Metastatic Colonization Pinpoints Cellular Mechanisms of Circulating Tumor Cell Extravasation

**DOI:** 10.3389/fonc.2021.641187

**Published:** 2021-09-23

**Authors:** Tyler A. Allen, Mark M. Cullen, Nathan Hawkey, Hiroyuki Mochizuki, Lan Nguyen, Elyse Schechter, Luke Borst, Jeffrey A. Yoder, Jennifer A. Freedman, Steven R. Patierno, Ke Cheng, William C. Eward, Jason A. Somarelli

**Affiliations:** ^1^ Duke Cancer Institute, Duke University Medical Center, Durham, NC, United States; ^2^ Department of Molecular Biomedical Sciences and Comparative Medicine Institute, North Carolina State University, Raleigh, NC, United States; ^3^ Department of Population Health and Pathobiology, College of Veterinary Medicine, North Carolina State University, Raleigh, NC, United States; ^4^ Department of Medicine, Division of Medical Oncology, Duke University Medical Center, Durham, NC, United States; ^5^ Joint Department of Biomedical Engineering, University of North Carolina at Chapel Hill and North Carolina State University, Chapel Hill, NC, United States; ^6^ Department of Orthopedics, Duke University Medical Center, Durham, NC, United States

**Keywords:** angiopellosis, circulating tumor cell cluster, tumor cell extravasation, cancer exodus hypothesis, metastasis, osteosarcoma

## Abstract

Metastasis is a multistep process in which cells must detach, migrate/invade local structures, intravasate, circulate, extravasate, and colonize. A full understanding of the complexity of this process has been limited by the lack of ability to study these steps in isolation with detailed molecular analyses. Leveraging a comparative oncology approach, we injected canine osteosarcoma cells into the circulation of transgenic zebrafish with fluorescent blood vessels in a biologically dynamic metastasis extravasation model. Circulating tumor cell clusters that successfully extravasated the vasculature as multicellular units were isolated under *intravital* imaging (n = 6). These extravasation-positive tumor cell clusters sublines were then molecularly profiled by RNA-Seq. Using a systems-level analysis, we pinpointed the downregulation of KRAS signaling, immune pathways, and extracellular matrix (ECM) organization as enriched in extravasated cells (p < 0.05). Within the extracellular matrix remodeling pathway, we identified versican (*VCAN*) as consistently upregulated and central to the ECM gene regulatory network (p < 0.05). Versican expression is prognostic for a poorer metastasis-free and overall survival in patients with osteosarcoma. Together, our results provide a novel experimental framework to study discrete steps in the metastatic process. Using this system, we identify the versican/ECM network dysregulation as a potential contributor to osteosarcoma circulating tumor cell metastasis.

**Graphical Abstract:**
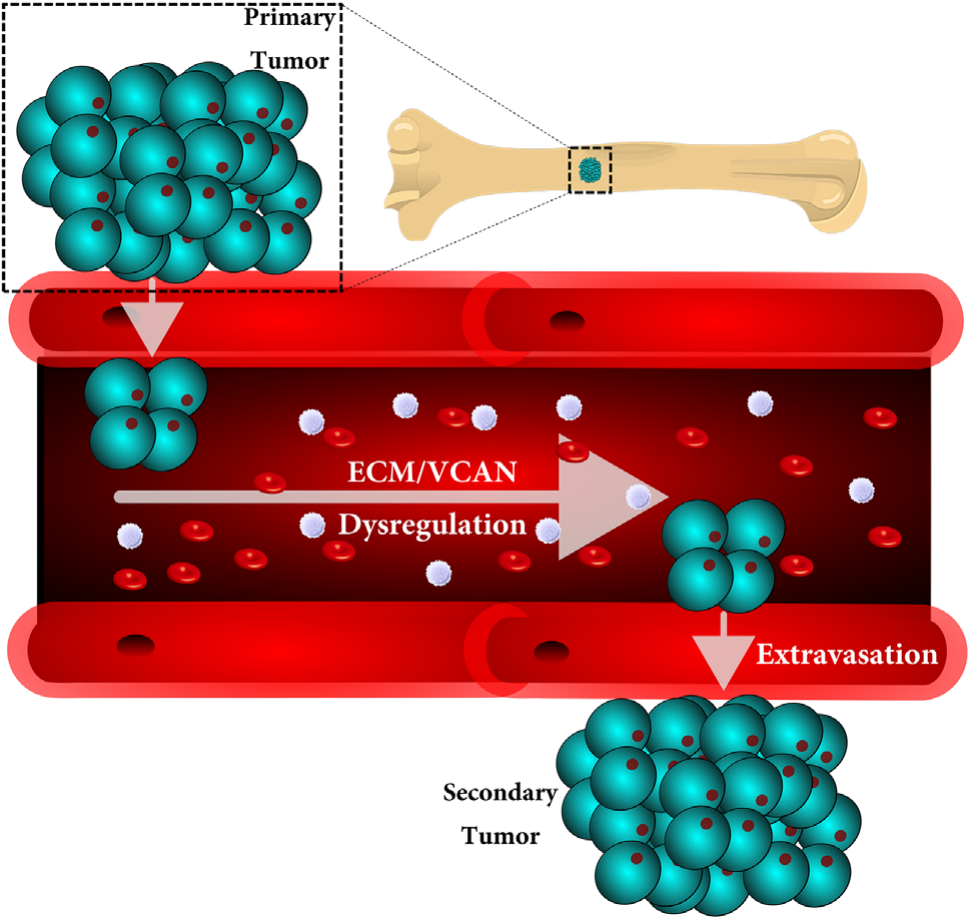
A graphical abstract of the proposed findings this study identified.

## Introduction

Metastasis is a complex process, involving multiple genetic, epigenetic, biochemical, and physical changes in cancer cells and their associated microenvironments. Although metastasis accounts for the majority of all cancer-related deaths, accumulating data suggests that the majority of metastasis is a result of circulating tumor cells (CTC) clusters, rather than individual tumor cells (1–7). These CTC clusters show an increased ability: 1) to spread throughout the body, 2) withstand the hemodynamic forces of the blood stream, 3) and proliferate once they extravasate at distant sites (8, 9). Additionally, studies have revealed that cell clusters exit circulation through the recently identified extravasation mechanism, known as angiopellosis, in which the blood vessels actively remodel, allowing the clusters to exit while maintaining their multicellularity (5, 10–14). The maintenance of this multicellularity has been shown to provide distinct survival and proliferative advantages, and it has been previously proposed that CTC clusters may be responsible for the majority of cancer metastasis (3, 4, 6, 15, 16).

Utilizing this system, we set out to establish a model to better identify and understand the molecular drivers of extravasation in metastatic osteosarcoma. Osteosarcoma (OS) is a rare, but aggressive primary bone cancer that predominantly affects children and young adults. It is the most common primary bone cancer, and the 5-year survival for patients who present with metastatic disease is, at best, 30% (17–20). Sadly, there have been no new treatments for metastatic OS in the past three to four decades, and an improved understanding of OS metastasis is urgently needed to identify the molecular mechanisms and pinpoint new potential therapeutic targets to prevent metastatic spread (21, 22). Here, we used a zebrafish model of metastasis to infuse fluorescent canine OS cells into Tg(fli1a:egpf) zebrafish with fluorescent blood vessels (5, 10, 23) to test the hypothesis that CTC clusters would exhibit unique molecular profiles associated with their ability to migrate to distant sites and extravasate. Zebrafish vasculature has previously been shown to be a suitable model system to understand the vascular environments in mammals, including humans (12–14, 24–26). The establishment of this model can be used to further identify and study targets or markers involved in the extravasation process.

We used *intravital* imaging to observe CTCs extravasating as clusters. These clusters were then isolated, expanded, and characterized by RNA-Seq. These efforts identified several key pathways commonly enriched in human and canine OS extravasated clusters, including the downregulation of KRAS signaling, interferon gamma response, and other immune pathways, and extracellular matrix remodeling. Among the genes commonly altered in both extravasation models, we identified versican, an extracellular matrix proteoglycan, as a potential mediator of CTC cluster extravasation. Versican, a large aggregating chondroitin sulfate proteoglycan, is an important ECM component associated with tumorigenesis (27, 28). Previous *in vitro* and *in vivo* studies have shown that versican enhances cancer cell survival, growth, migration, invasion, angiogenesis, drug resistance, and metastasis and has been shown to induce malignancy in OS cells through the interaction with TGF-beta (29–33). Additionally, an increased expression of versican has been reported in several types of malignancies including brain tumors, leukemia, breast, prostate, colon, lung, and ovarian cancers, and is generally associated with a poor prognosis (30, 32, 34–37). Our collective data suggest that a combination of *intravital* imaging and molecular characterization in a zebrafish model of metastasis can be used to identify the molecular mechanisms and gene regulatory pathways involved in discrete steps in the metastatic process.

## Materials and Methods

### Animals

All experiments involving live zebrafish were performed in accordance with relevant institutional and national guidelines and regulations and were approved by the North Carolina State University Institutional Animal Care and Use Committee. For zebrafish, the transgenic lines *Tg(fli1a:EGFP)* were used in this study. The resulting embryos were screened at 48 hpf for the expression of transgene expression. In order to prevent pigmentation, 0.2 mM N-phenylthiourea (PTU; Sigma) was applied to all embryos starting at 24 hpf.

### Embryo Preparation and Tumor Cell Implantation

Dechorionized 48 hpf zebrafish embryos were anesthetized with 0.004% tricaine (Sigma) and positioned on a 200 mm x 15 mm Petri dish coated with 3% agarose. Canine osteosarcoma cells were trypsinized into single cell suspensions, resuspended in phosphate-buffered saline (PBS; Invitrogen), kept at room temperature before implantation, and implanted within 2 hours. Any non-fluorescent cells were labelled with the fluorescent cell tracker DiI (Invitrogen) according to the instructions of the manufacturer. The cell suspension was loaded into borosilicate glass capillary needles (1 mm o.d. × 0.78 mm i.d.; World Precision Instruments) and the injections were performed using a PV830 Pneumatic Pico pump and a manipulator (WPI). A total of 30–100 cells, were injected at approximately 50 μm above the ventral end of the duct of Cuvier where it opens into the heart. The approximate injection parameters were: injection pressure =300 p.s.i., holding pressure = 10 p.s.i., injection time = 0.2 seconds. Injected tumor cells could normally be seen entering the vasculature 15–30 minutes after injection and starting to arrest in the vessels of the tail as clusters within 2 hours after injection. After implantation with mammalian cells, zebrafish embryos (including non-implanted controls) were maintained at 32°C. Normally, cell injected embryos were euthanized at the end of experiments (~72 hpf) by tricaine overdose. For each cell line or condition, data are representative of ≥three independent experiments, with ≥5 embryos/group. Experiments were discarded when the survival rate of the control group was <80%.

### Zebrafish Embryo Preparation and Microscopy

For live imaging in the light-sheet microscope, 48 hpf zebrafish embryos were anesthetized using 0.016% tricaine (Sigma) and then were embedded in 1.3% low-melting-temperature agarose (Sigma; prepared in filtered fish facility water) inside a glass capillary [1.5/2.0-mm inner/outer diameter, 20-mm length (Zeiss)]. The embryos were centered in the capillary and oriented. After gel formation, the section of the agarose cylinder containing the tail of the embryo was extruded from the capillary by inserting wax into the capillary on the side opposite to the fish. The sample chamber of the light-sheet microscope was filled with filtered fish facility water, and the capillary was inserted for imaging. Specimens were maintained at 32°C throughout the imaging period. Fluorescent image acquisition was performed using a Zeiss Lightsheet Z.1. Z-stacks were processed for maximum intensity projections with the Zeiss ZEN software. For timelapse (4D) images, zstacks were taken every 5–15 minutes for a total time of up to 24 hours with a step number between 50 and 200 and step size of 0.3–2.0 μm. Images were adjusted for brightness and contrast using the Zeiss ZEN Software. Confirmation of injected cell migration from inside of the lumen to surrounding tissue was done using the Zeiss ZEN software 3D retendering capability.

### Cell Culture

Canine osteosarcoma cell lines: D17 and HMPOS were cultured in IMDM/10% (v/v) fetal bovine serum/2 mM L-glutamine/100 U/ml penicillin/100 μg/ml streptomycin (all Life Technologies, Germany). All cell lines were cultured at 37°C and 5% (v/v) CO_2_. All lines were authenticated and tested negative for mycoplasma contamination.

### Isolation and Culturing of Injected Tumor Cells From Zebrafish Embryos

Following confirmation of extravasation of injected tumor cells, the embryos section containing the extravasated cells were dissected within 24 hours post injection. Dissected embryos sections were transferred to phosphate-buffered saline (PBS) containing 50 U/mL penicillin (Gibco) and 0.05 mg/mL streptomycin (Gibco) (PBS/PS) for at least 15 min. The PBS/PS solution was refreshed once and individual embryos were transferred into a sterile tube (embryo + 500 uL PBS/PS) for 5 minutes. Embryos were then transferred to 200 uL of a 1% bleaching solution for 5 min. After replacing the bleaching solution with PBS/PS immediately and incubating for 5 min, embryos were centrifuged at 1,200 g (rcf) for 2 min at room temperature and the supernatant was discarded. Next, we added 300 uL of TripLE (Gibco) and incubated for 45 minutes at 37°C in a thermomixer, while mixing at 800 rpm. Next, we pipetted the embryo-TripLE, with a 200-uL tip, several times up and down under sterile conditions (cell culture hood), and immediately centrifuged (4 min, 1,200 g at room temperature). We discard the supernatant and resuspended with 400 uL of PBS and centrifuge (4 min, 1,200 g at room temperature). We resuspended the cell pellets in 200 uL of growth media and transferred the cell suspension to a 96-well plate (200 ul of media per well).

### RNA-Seq Analysis

Following isolation from the zebrafish and the *in vitro* establishment of extravasation-positive cell lines, total RNA was purified with the miRNeasy extraction kit (Qiagen) according to the manufacturer guidelines from cell pellets. RNA quality was assessed on a Bioanalyzer 2100 instrument using the RNA 6000 Nano Kit (Agilent). mRNA-seq sequencing libraries were prepared from 1 µg of purified RNA using the Illumina’s TruSeq Stranded mRNA Library Prep Kit. Deep sequencing was performed on a Nextseq500 sequencer (Illumina) using 75 bp paired-end reads. Raw BCL (base call) files generated from the NextSeq sequencer were converted to FASTQ files using the bcl2fastq Conversion Software v2.18. During BCL to FASTQ processing, bcl2fastq also separates multiplexed samples, removes adapters, trims low quality bases, and removes low quality reads. Raw RNA-seq data in the FASTQ file format was quality controlled during and after sequencing to identify the potential technical issues. Cleaned sequencing reads were then mapped to the canine reference genome using STAR to generate read counts for each of the annotated genes (38). Gencode transcript annotations were supplied to facilitate the mapping of reads spanning known splicing junctions. The raw gene read count data was normalized using the voom approach (39). The differential expression analysis was performed using the linear model approach provided by the limma package (40). For the differential expression analysis, we only kept those genes with more than 30 raw read counts in at least two biological samples. The extravasation-positive D17 and HMPOS cells were analyzed against wild-type D17 and HMPOS cells grown *in vitro* without introduction to the zebrafish environment. The p-values for the coefficient/contrast of interest were adjusted for multiple testing using the Benjamini and Hochberg’s method (41), which controls the expected false discovery rate (FDR). The significance threshold for gene differential expression was defined as a fold change greater than 2 and FDR less than 0.05. Venn diagrams were generated in Biovenn (42). Pathway analysis was performed using gene set enrichment analysis (43). Gene regulatory networks were inferred and annotated using GeneMANIA (44) within Cytoscape v3.8. For the pathways of interest identified by gene set enrichment analysis (Reactome extracellular matrix organization, Hallmark KRAS signaling UP, Hallmark IFNγ response), the list of genes in each pathway was obtained from MSigDB and used as an input for GeneMANIA. Networks were annotated using the log2 fold change from the RNA-Seq data for each cell line. The Cytoscape network analyzer tool was used to infer network interaction parameters, and a sum rank statistic was created from the following parameters: degree, clustering coefficient, closeness, betweenness, neighborhood connectivity, and stress.

### Data Availability

All RNA-Seq data is available at NCBI GEO, Accession: GSE164246.

### Statistical Analysis

Parametric and nonparametric analyses were used throughout the study. For continuous distribution data sets, we used either the Student’s t-test or the Mann–Whitney Wilcoxon test for two groups. For multiple groups, either ANOVA followed by Fisher least square difference *post hoc* test or the Kruskal–Wallis followed by the Mann–Whitney–Wilcoxon was used. For two groups characterized as a frequency (percentage), we used comparison of proportions. P-values are presented either in the figure legend or figure panels.

The R2 genomics web tool (https://hgserver1.amc.nl/cgi-bin/r2/main.cgi) was used to plot Kaplan Meier curves to analyze the prognostic value of *VCAN* expression in osteosarcoma patients. The settings for this analysis were “Kaplan scan a single gene” and using the default time cut-offs. These data are from a previously published study and included 88 osteosarcoma patients, the clinical characteristics of whom are described in the originally-published study (PMID: 22454324; GEO accession number: GSE33383). Briefly, 64.3% of the patients in this cohort were male, 34.5% were female, and 1.2% with unknown gender. A total of 76.2% of the patients were under 20 years of age, 22.6% were over 20 years of age, and 1.2% (1) of the patients have unknown age. Primary tumor locations included the femur (47.6%), tibia/fibula (33.3%), humerus (13.1%), axial skeleton (1.2%), and unknown/other (4.8%). Histological subtype included osteoblastic (61.9%), chondroblastic (10.7%), fibroblastic (8.3%), telangiectatic (4.8%), minor subtype (13.1%), and unknown (1.2%). A total of 45.2% of the patients were grade 1 or 2, 39.3% were grades 3 or 4, and 16.7% were unknown or with tumor grades not available. Among the cohort, 82.1% of the patients had no metastasis at diagnosis, 16.7% had metastasis at diagnosis, and 1.2% had an unknown status of metastasis at diagnosis (45).

## Results

### Circulating Canine Osteosarcoma Cells Extravasate as Clusters Through Angiopellosis

**Figure 1:**
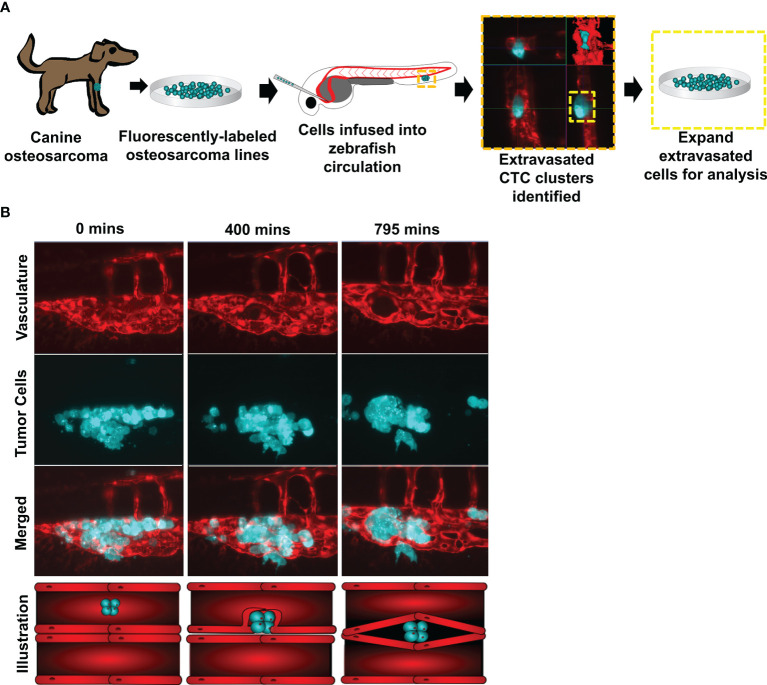
Circulating canine osteosarcoma clusters extravasate by angiopellosis. **(A)** Illustration of the project workflow. Canine osteosarcoma cell lines were fluorescently labeled and infused into the circulation of *Tg(fli1a:egpf*) with fluorescent blood vessels. Tumor cells which extravasated as clusters were isolated and expanded, and these sub-lines were then molecularly profiled. **(B)** Representative image of infused D17 tumor cells aggregating as clusters in zebrafish circulation. Tumor cell clusters begin to extravasate through the endothelial cells of the blood vessels during metastasis. The tumor cluster is eventually completely removed from the inside of the vessels and is lodged in an extravascular cavity while maintaining multicellularity and not disassociating into single cells. SB = 20µM.

Two fluorescently labeled canine osteosarcoma cell lines (HMPOS and D17) were infused into the circulation of *Tg(fli1a:EGFP)* zebrafish with fluorescent blood vessels. We observed and isolated three biological replicates of the extravasated cell clusters (n = 3) (**Figure 1A**). Following cell infusion, zebrafish were microscopically observed for up to 48 hours. The clusters extravasated using angiopellosis, maintaining multicellularity, as noted by the characteristic remodeling of the vasculature around the clusters until the successful exit into the extravascular tissue (**Figure 1B** and **Supplementary Video 1**) (5, 10, 24).

### A Systems-Level Approach Identifies Differential Expression of Core Pathways Involved in Proliferation, Immune Surveillance, and Extracellular Matrix Remodeling

**Figure 2:**
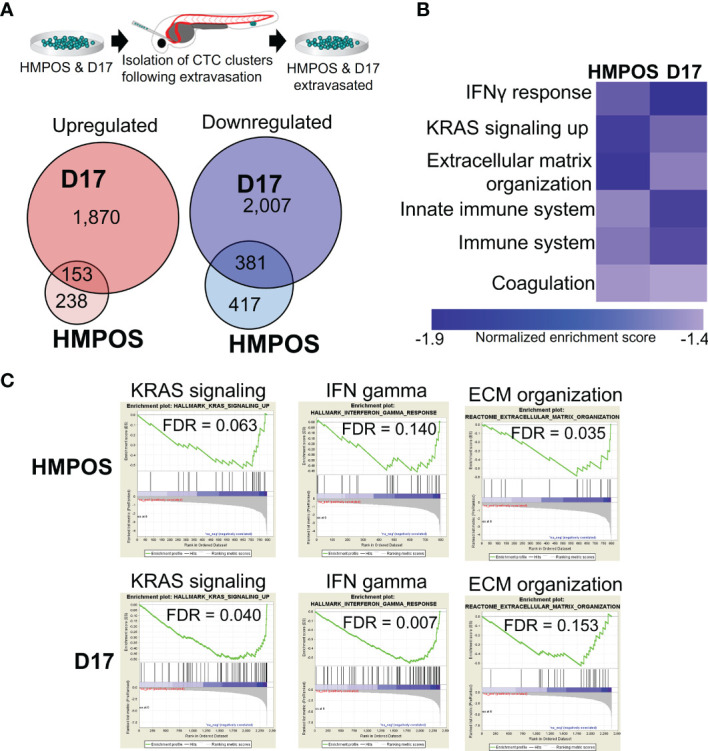
Extravasated circulating tumor cells display differential expression of genes and pathways. **(A)** Overlaps in the upregulated and downregulated genes in extravasated circulating tumor cells. **(B)** Heat map of pathways commonly downregulated in extravasated circulating tumor cells. **(C)** Enrichment plots for KRAS signaling, IFN gamma response, and extracellular matrix (ECM) organization in HMPOS and D17 extravasated cells.

To understand the molecular mechanisms by which tumor cell clusters successfully extravasated, we isolated the extravasation-positive clusters of HMPOS (a highly metastasizing sub-line of the POS osteosarcoma line) (46, 47) and a second metastatic osteosarcoma line, D17 (48), and performed RNA Sequencing (RNA-Seq) on the resulting sub-lines (**Figure 2A**). Analysis of the RNA-Seq data from extravasated osteosarcoma cells revealed 391 (HMPOS) and 2,023 (D17) upregulated genes and 798 (HMPOS) and 2,338 (D17) downregulated genes (p < 0.05) (**Figure 2A** and **Supplementary Figure 1**). To identify the pathways that are commonly altered in the extravasated cell clusters, we applied a systems-level analysis of the RNA-Seq data. We first used gene set enrichment analysis to identify the pathways that were positively and negatively enriched in extravasated cells. While no positively-enriched pathways were common to both cell lines, the pathways enriched in downregulated genes in extravasated cells from both HMPOS and D17 lines included the KRAS signaling, interferon gamma response and other immune response pathways, and extracellular matrix organization (**Figures 2B, C**).

Next, we constructed gene regulatory networks for the enriched pathways from extravasated HMPOS and D17 cells. Gene regulatory networks provide a visual representation of gene interaction pathways, with the genes represented as circular nodes and the interactions between genes represented by lines (edges). Networks were visualized by overlaying the RNA-Seq log2 fold change values for each gene in the pathway, with larger red nodes representing a higher mRNA upregulation and larger blue nodes representing a larger mRNA downregulation in extravasated cells (**Supplementary Figures 2**, **3**). Qualitatively, these networks reveal largely unique patterns between the two cell lines, with distinct subsets of genes from unique locations within the network altered in each cell line (**Supplementary Figures 2**, **3**). To provide a more quantitative understanding of these differences between the cell lines, we next applied network analysis to these pathways, which outputs a suite of quantitative metrics to define the overall connectedness of the gene regulatory network. Networks were analyzed for the following parameters: degree, clustering coefficient, closeness, betweenness, neighborhood connectivity, and stress. We then plotted the sum rank score of these network parameters by the log2 fold change for each gene with a significant up- or downregulation in the network (**Supplementary Figures 2**, **3**). These analyses show an overall downregulation of nodes across a range of connectivity within the network for both cell lines, with D17 cells having more downregulated genes in the network (**Supplementary Figures 2**, **3**). Despite the differences between the cell lines, several mRNA alterations with a high network connectedness were commonly altered in both cell lines (**Supplementary Figures 2**, **3**).

### Extravasated Cells Upregulate Versican, Which Contributes to Extracellular Matrix Remodeling and Cellular Migration

**Figure 3:**
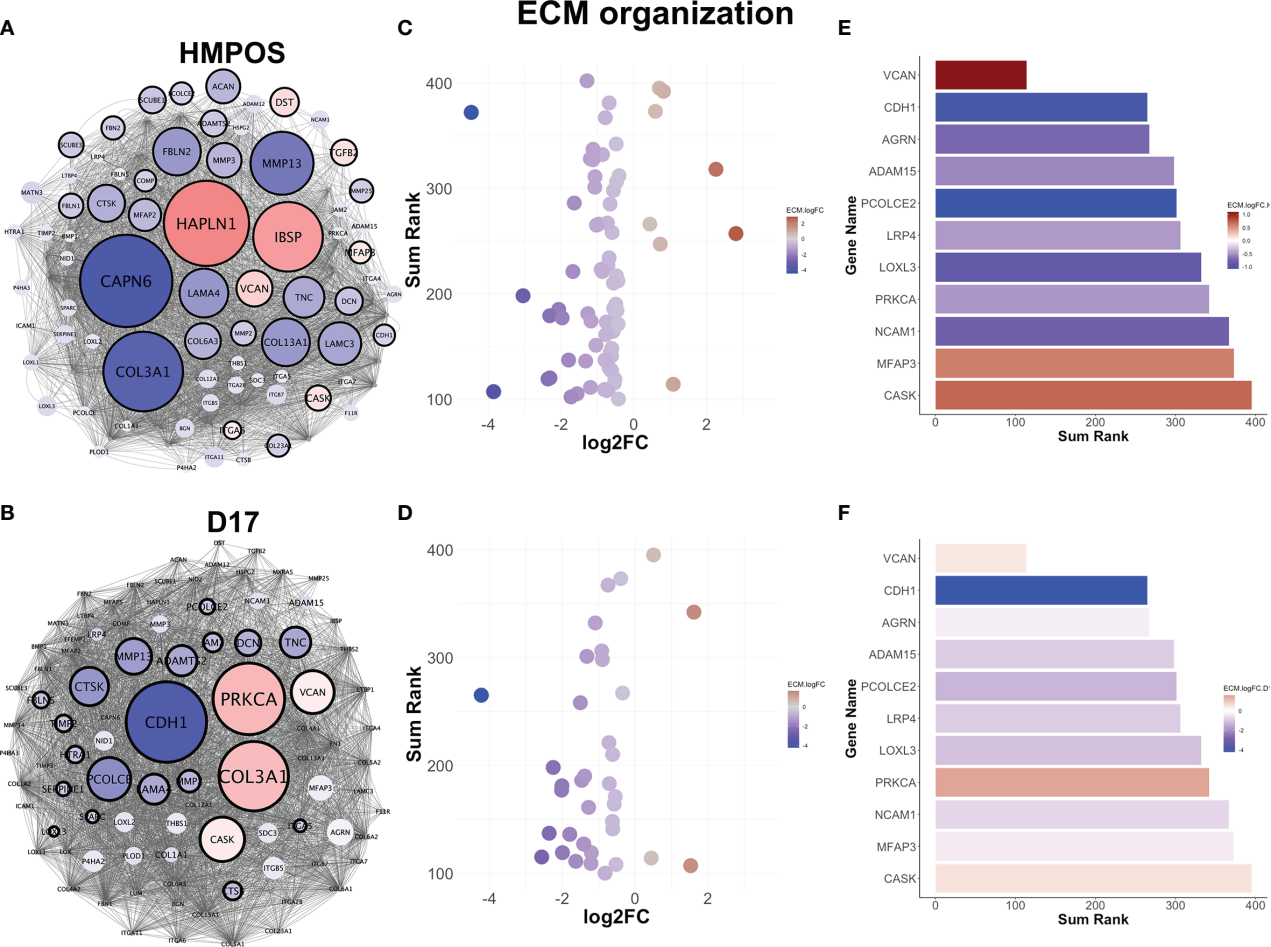
Extravasated circulating tumor cells downregulate extracellular matrix remodeling. **(A)** Gene regulatory networks for the extracellular matrix remodeling pathway in HMPOS and **(B)**. D17. Blue denotes downregulation and red denotes upregulation (FDR < 0.05). Nodes are scaled proportionally to log2FC, with larger nodes depicting greater log2FC between extravasated and parental cell lines. **(C)** Scatter plot of sum rank of network connectivity parameters by log2FC for HMPOS and **(D)** D17. **(E)** Bar graphs of top connected genes in the pathway, colored by log2FC for HMPOS and **(F)** D17.

We focused next on the extracellular matrix remodeling pathway as a known phenotypic driver of migration/invasion and metastasis. Like the other gene regulatory networks, extracellular matrix remodeling is commonly downregulated in both HMPOS and D17 extravasated cells (**Figures 3A, B**), but displays largely-unique gene-level changes in each cell line (**Figures 3A, B**). Most of the genes in the network are downregulated, with a very few upregulated highly-connected genes in the network (**Figures 3C, D**). One of the few commonly-altered genes in both cell lines, however, is versican. Versican is an extracellular matrix proteoglycan that regulates matrix remodeling, migration, and invasion (49). Interestingly, versican is significantly upregulated in both HMPOS and D17 extravasated cells and among the most connected genes in the ECM organization pathway (**Figures 3E, F**).

**Figure 4:**
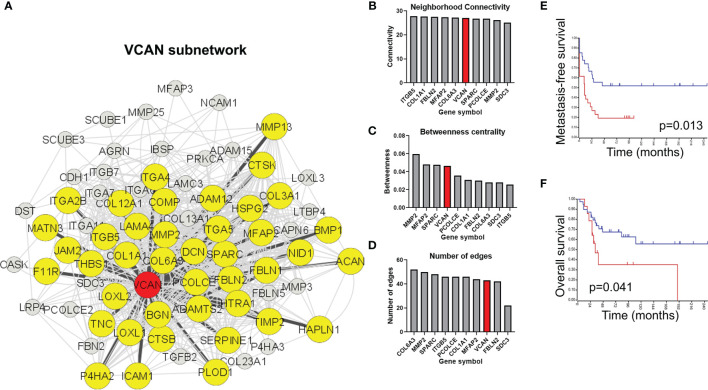
Versican-mediated extracellular matrix organization is enriched in extravasated circulating tumor cells. **(A)** Versican is commonly upregulated in both cell lines and interacts with a core subset of the extracellular matrix organization pathway. Yellow nodes indicate proteins that share at least one interaction with versican. **(B)** Ranking of top nodes based on neighborhood connectivity, **(C)** betweenness centrality, and **(D)** number of edges in the extracellular matrix remodeling pathway. Prognostic value of VCAN for **(E)** metastasis-free survival and **(F)** overall survival in osteosarcoma patients.

Analysis of the extracellular matrix remodeling network indicates that versican interacts with 58% of the core extracellular matrix remodeling network (42/72 nodes) within the pathway (**Figure 4A**, yellow nodes). Indeed, versican is in the top 10 most interconnected members of the network for neighborhood connectivity, betweenness centrality, and number of edges (**Figures 3B–D**). Together, these analyses indicate that versican is commonly upregulated in extravasated cells and interacts with a majority of nodes in the extracellular matrix remodeling pathway.

Versican was ranked in the top nodes based on neighborhood connectivity, betweenness centrality, and number of edges in the extracellular matrix remodeling pathway (**Figures 4B–D**). Also consistent with a role in extravasation and metastasis, an elevated versican expression is prognostic for a poorer metastasis-free survival (**Figure 4E**) and overall survival in osteosarcomas (**Figure 4F**).

## Discussion

The ability of CTCs to survive in circulation, extravasate at distant sites, and form new tumors is extremely rare (3, 50); however, when successful, these events often represent the precursors to a deadly disease (1, 51–53). During dissemination, CTCs must endure harsh conditions, including novel microenvironments, exposure to different cell types and signals, immune targeting, anchorage-independent growth, and shear force from circulation (1, 3, 6, 8, 12). Studies have shown that when CTCs form clusters, their ability to survive the metastatic process and seed distant sites is markedly increased. Some have postulated that the majority of cancer metastases occur as a result of these clusters rather than by individual CTCs (1–3, 50). The mechanisms through which these CTC clusters extravasate are only beginning to be understood. For example, melanoma and cervical CTC clusters have been observed extravasate out of blood vessels through *angiopellosis*, in which endothelial cells remodel the vessel architecture around the CTC clusters (5, 10, 12, 50, 54). This allows CTC clusters to maintain their multicellular phenotype (5). The establishment of relevant models to pinpoint and further investigate the specific markers of extravasation are limited, but this zebrafish model serves as a unique method to leverage the ability to perform *intravital* imaging and subsequent cell isolation to interrogate this process more fully (2, 3, 8, 13, 25, 27, 51).

Canine OS cells were specifically used in this project to 1) identify targets for further validation in human cells and 2) to leverage companion animal cancer data to investigate metastasis in both canines and humans. Many biologic behaviors of OS are conserved between people and dogs, and evidence suggests that leveraging the power of cross-species analyses facilitates the understanding of fundamental drivers of cancer mechanisms, as well as factors contributing to cancer initiation and progression (55–58). The ability of tumor cells from varying species and cancer types to undergo angiopellosis suggest that this mechanism of extravasation may be a common feature of CTC clusters. Prior studies have shown that non-tumor cells and cell membrane-coated microparticles possess the ability to undergo *angiopellosis*, suggesting that this phenomenon is not exclusive to CTCs, but may be utilized by CTCs during the metastasis process. Our data have pinpointed potential molecular drivers of extravasation, including the downregulation of proliferative signals (e.g., KRAS signaling), immune evasion, and versican-mediated ECM dysregulation during the metastasis process. Angiopellosis may serve as a physiological mechanism hijacked by specific types of cancer cells allowing for extravasation, similar to other extravasation mechanisms like cancer-mediated necrosis of endothelial cells, and transmigratory cup formation in neutrophil extravasation (59, 60). Further studies are needed to explore whether other non-cancerous cells utilize a similar gene dysregulation during *angiopellosis*.

Consistent with our analyses of osteosarcoma patient data, prior studies have shown that elevated levels of versican are associated with poor prognosis for patients with a wide range of malignant tumors (28, 30, 36). Indeed, versican and other ECM proteoglycans have been shown to impact the tumor cell behavior in myriad cancer types, in both human and canine models (61). While the exact mechanism by which versican promotes OS progression remains to be fully elucidated, previous studies suggest that versican contributes to the formation of a macromolecular complex in the ECM, which may account for the increased invasion and metastasis through promoting cancer cell motility and increased cell-cell adhesion (28, 62, 63).

In addition to its role in cellular migration, it is also possible that versican acts by altering cell adhesion, which contributes to the maintenance of tumor-propagating-like features of CTC clusters and augments their ability to proliferate at distant sites. Consistent with this, the cluster phenotype in CTCs is known to induce cells to express a more stem cell-like biology through the DNA methylation of binding sites typically occupied by stemness and proliferation regulators, including *OCT4*, *NANOG*, *SOX2*, and *SIN3A* (6, 64–67). Interestingly, the clustering of stem cells has been shown to protect their active pluripotent pathways with the disruption of cell-cell junctions resulting in the downregulation of proliferation regulators coupled with loss of stemness features (68–71). Previous studies have shown that the rates of CTC extravasation as either clusters or individual cells is similar, and while both could exit the vasculature similarly, the CTC clusters showed an increased ability to continue proliferation once out of the circulation, which contributed to their increased metastatic potential (2, 5). In this context, the dysregulation of versican and ECM may play an integral role in the cluster ability of CTCs by granting them an increased ability to form and maintain clusters, which gives them an increased chance to form tumors following extravasation. Additionally, versican-mediated alterations in cell adhesion may contribute to the interaction and adhesion of CTCs to the endothelial layer, which is known to play an important role in extravasation. Further studies are needed to fully elucidate 1) the impact of versican on CTC cluster formation and maintenance, 2) its localization during this process, and 3) its impact on CTC endothelial layer interactions/adhesion.

One unique advantage of this extravasation model system is that we can directly observe, capture, and molecularly interrogate CTC clusters exiting the vasculature. Current limitations in oncological imaging and detection normally prevent tumors from being isolated/identified unless they are still in the circulation or not until the CTCs have already extravasated and formed detectable tumor masses (4, 15, 51, 72). The need to address the specifics of CTC cluster metastasis/extravasation is paramount, as the cluster phenotype exhibits a markedly higher metastatic potential over their individual cell counterparts (1, 2, 5). The present study attempts to address some of these limitations and improve our understanding of the molecular underpinnings of CTC cluster extravasation.

This study had several limitations. The RNA-Seq analysis was performed on clusters that were isolated from zebrafish, but the control cells for this analysis were the parental cells, instead of un-extravasated cells exposed to the zebrafish circulation. Despite multiple attempts to isolate these cells, the non-extravasated cells were not able to be successfully isolated, due to the reliance of the isolation system on the cells of interest being outside of the vasculature; a common limitation in this type of study (2, 11, 73–76). In addition, we were not able to obtain microscopic images to clearly reveal the level of detail necessary to confirm the extravasation of CTC clusters by angiopellosis. This study was instead focused on isolating the extravasated cells for downstream processing. Furthermore, to achieve sufficient levels of RNA for analysis, cells were expanded following isolation, rather than sequenced immediately following isolation, which serves as an additional variable in the RNA-seq results. Another limitation is the possibility that versican upregulation is a part of global dysregulation and regulated in conjunction with other ECM genes by an upstream regulator. Future studies will be needed to further investigate this potential, but also serves as a promising direction to better understand the global role of ECM in cancer progression.

This study focused on the molecular profiling of the CTC clusters that extravasated, rather than individual CTCs transiting the circulation. Due to this, it is important to note that the profiles identified may be exclusive to the CTC clusters that extravasate, rather than all CTCs. Overall, the observation of VCAN dysregulation in the canine OS cell lines used provide a starting point for more complex *in vivo* studies using this model to understanding the dynamics of extravasation and metastasis. Future studies are needed to investigate differences in the extravasation potential across different OS models and characterize their cluster morphology and underlying genomics. Additionally, future studies will examine a panel of high, low, and non-metastatic OS cell lines to determine the difference in the behavior and gene expression between cancers of varying metastatic/extravasation potential.

In summary, this study establishes the zebrafish *intravital* imaging model as a means to further investigate the specific steps of the extravasation process and identifies key pathway alterations that may drive extravasation during metastasis. These pathways may represent novel therapeutic targets to prevent CTC cluster formation, migration ability, and metastatic potential. Together, these data 1) establish a useful model to provide key insights into the biology of CTCs, extravasation, and metastasis, and 2) highlight an important connection between the phenotypic features of CTCs, including their ability to extravasate as multicellular clusters and their unique molecular features that may lead to the successful formation of secondary tumors.

## Data Availability Statement

The datasets presented in this study can be found in online repositories. The names of the repository/repositories and accession number(s) can be found below: NCBI GEO [accession: GSE164246].

## Ethics Statement

The animal study was reviewed and approved by the North Carolina State University Institutional Animal Care and Use Committee. Code: 13-083-B.

## Author Contributions

Conceptualization: TA. Methodology: TA, MC, and HM. Formal analysis: TA, HM, LN, ES, and JS. Investigation: TA, HM, LN, ES, and JS. Resources: TA, JS, WE, LB, JY, and KC. Writing—original draft preparation: TA. Writing—review and editing: TA, JS, WE, JF, SP, KC, and MC. Visualization: TA, JS, MC, LN, and HM. Supervision: TA, JS, and KC. Funding acquisition: TA, KC, JS, and WE. All authors contributed to the article and approved the submitted version.

## Funding

This research was funded by Andy’s Army Canine Cancer Awareness Project (to TA, HM), the Carter Chinnis Charitable Trust (to WE), and National Cancer Institute (F31 CA217153 to TA). The funders had no role in the design of the study; in the collection, analyses, or interpretation of data; in the writing of the manuscript, or in the decision to publish the results.

## Conflict of Interest

The authors declare that the research was conducted in the absence of any commercial or financial relationships that could be construed as a potential conflict of interest.

## Publisher’s Note

All claims expressed in this article are solely those of the authors and do not necessarily represent those of their affiliated organizations, or those of the publisher, the editors and the reviewers. Any product that may be evaluated in this article, or claim that may be made by its manufacturer, is not guaranteed or endorsed by the publisher.

## Supplementary Material

Supplementary Figure 1 Top differentially expressed genes compared to parental lines. **(A)** A bi-clustering heatmap visualizing the expression profile of the top 30 differentially expressed genes in the D17 cell line sorted by their p-value by plotting their FKPM expression values in each biological replicate/samples. **(B)** A bi-clustering heatmap visualizing the expression profile of the top 30 differentially expressed genes in the HMPOScell line sorted by their p-value by plotting their FKPM expression values in each biological replicate/samples.

Supplementary Figure 2 Extravasated circulating tumor cells downregulate KRAS signaling. **(A)** Gene regulatory networks for the KRAS signaling pathway in HMPOS and **(B)** D17. Blue denotes downregulation and red denotes upregulation (FDR<0.05). Nodes are scaled proportionally to log2FC, with larger nodes depicting greater log2FC between extravasated and parental cell lines. **(C)** Scatter plot of sum rank of network connectivity parameters by log2FC for HMPOS and **(D)** D17. **(E)** Bar graphs of top connected genes in the pathway, colored by log2FC for HMPOS and **(F)** D17.

Supplementary Figure 3 Extravasated circulating tumor cells downregulate the interferon gamma response pathway. **(A)** Gene regulatory networks for the interferon gamma response pathway in HMPOS and **(B)** D17. Blue denotes downregulation and red denotes upregulation (FDR<0.05). Nodes are scaled proportionally to log2FC, with larger nodes depicting greater log2FC between extravasated and parental cell lines. **(C)** Scatter plot of sum rank of network connectivity parameters by log2FC for HMPOS and **(D)** D17. **(E)** Bar graphs of top connected genes in the pathway, colored by log2FC for HMPOS and **(F)** D17.

Supplementary Video 1 A representative time-lapse video of circulating tumor cell clusters (cyan) extravasating from the inside/lumen of zebrafish blood vessels (red) into the surrounding extravascular cavity. SB = 20 µm.
