# Supplementary figures and images for: A Zebrafish Model of Metastatic Colonization Pinpoints Cellular Mechanisms of Circulating Tumor Cell Extravasation
Source: Front Oncol. 2021 Sep 23;11:641187. doi: 10.3389/fonc.2021.641187 (PMC8495265; doi:10.3389/fonc.2021.641187)

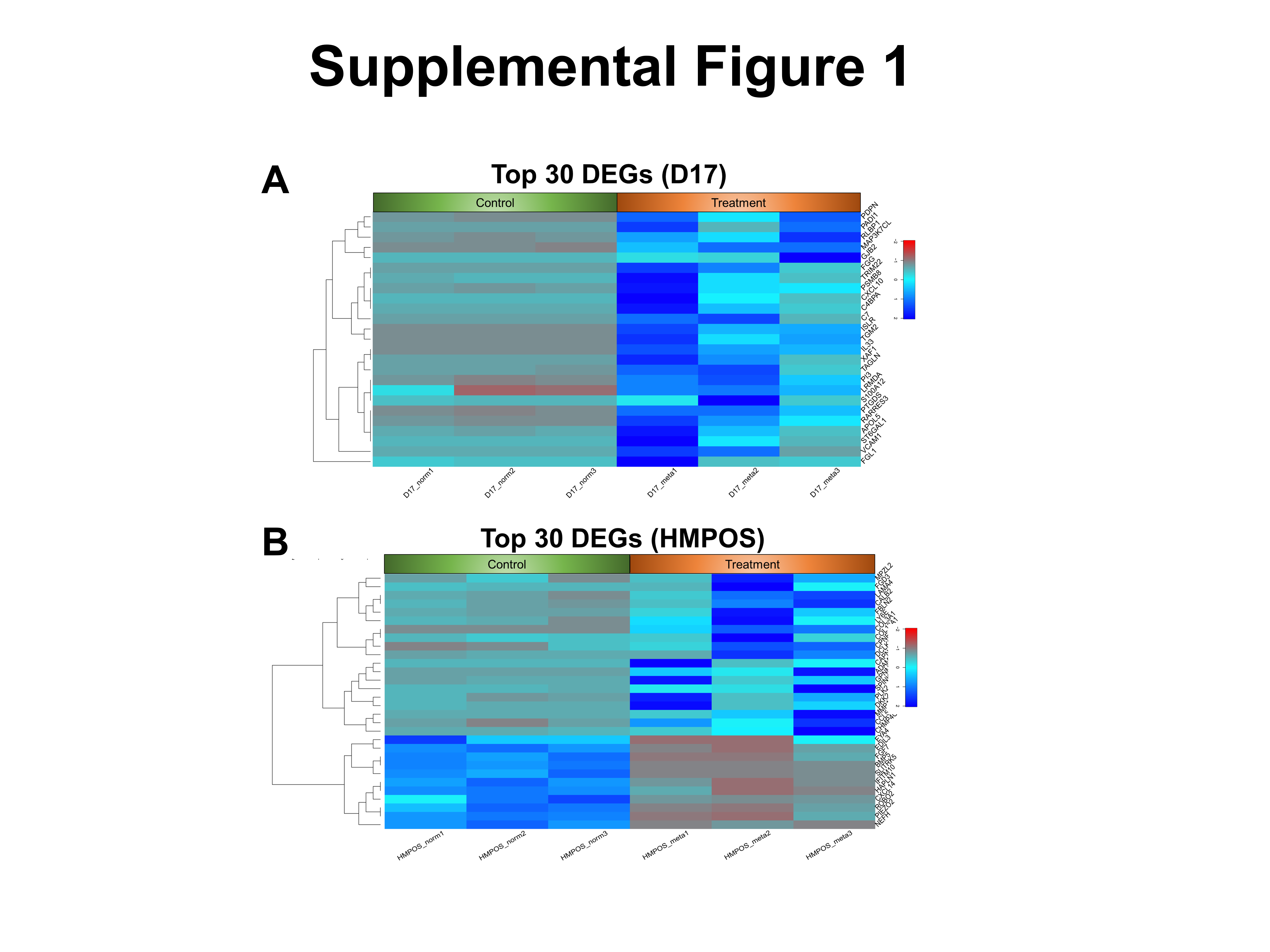

Supplement: Supplementary Figure 1 — Top differentially expressed genes compared to parental lines. (A) A bi-clustering heatmap visualizing the expression profile of the top 30 differentially expressed genes in the D17 cell line sorted by their p-value by plotting their FKPM expression values in each biological replicate/samples. (B) A bi-clustering heatmap visualizing the expression profile of the top 30 differentially expressed genes in the HMPOScell line sorted by their p-value by plotting their FKPM expression values in each biological replicate/samples. [file Image_1.tif]

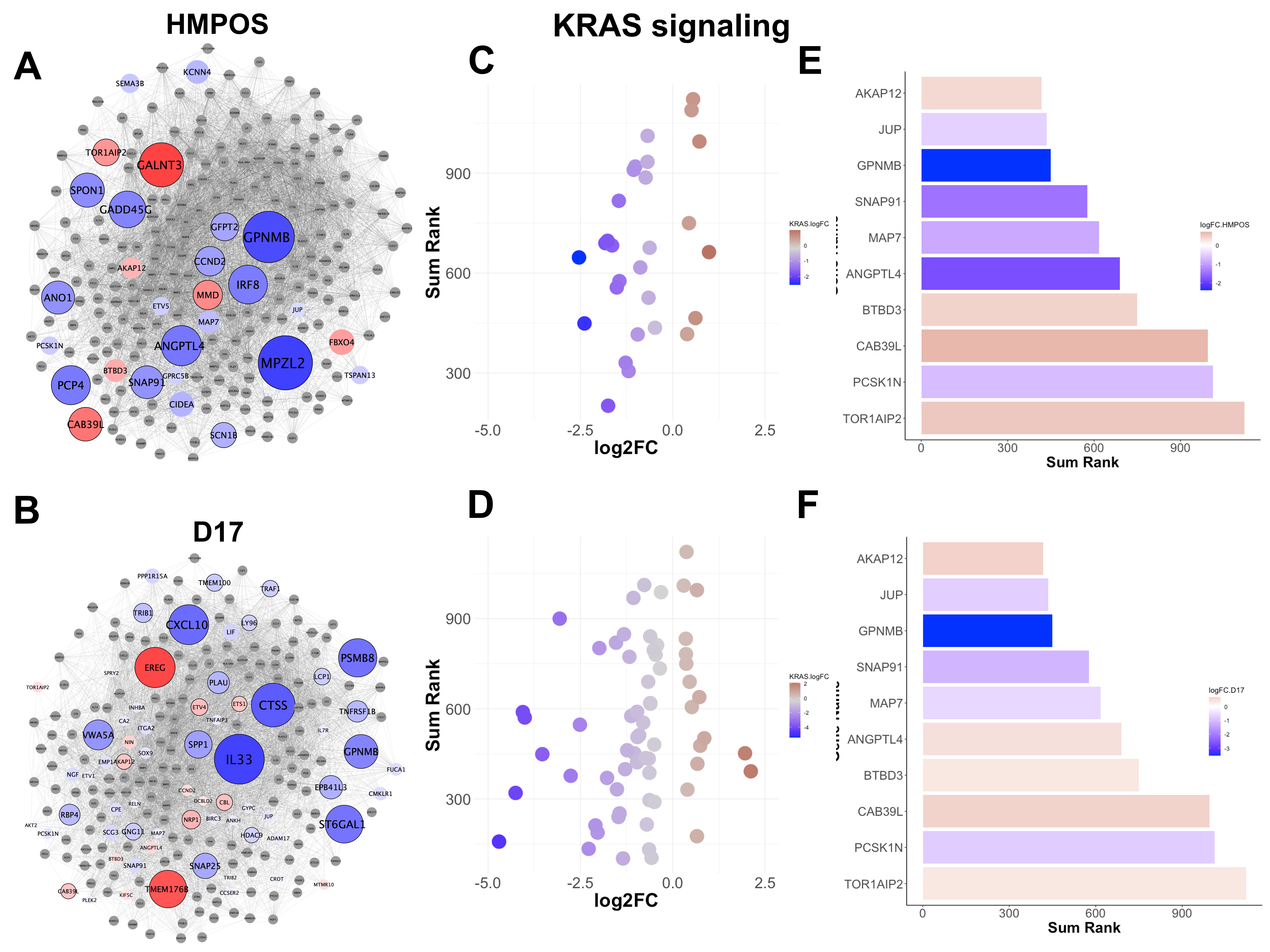

Supplement: Supplementary Figure 2 — Extravasated circulating tumor cells downregulate KRAS signaling. (A) Gene regulatory networks for the KRAS signaling pathway in HMPOS and (B) D17. Blue denotes downregulation and red denotes upregulation (FDR<0.05). Nodes are scaled proportionally to log2FC, with larger nodes depicting greater log2FC between extravasated and parental cell lines. (C) Scatter plot of sum rank of network connectivity parameters by log2FC for HMPOS and (D) D17. (E) Bar graphs of top connected genes in the pathway, colored by log2FC for HMPOS and (F) D17. [file Image_2.tif]

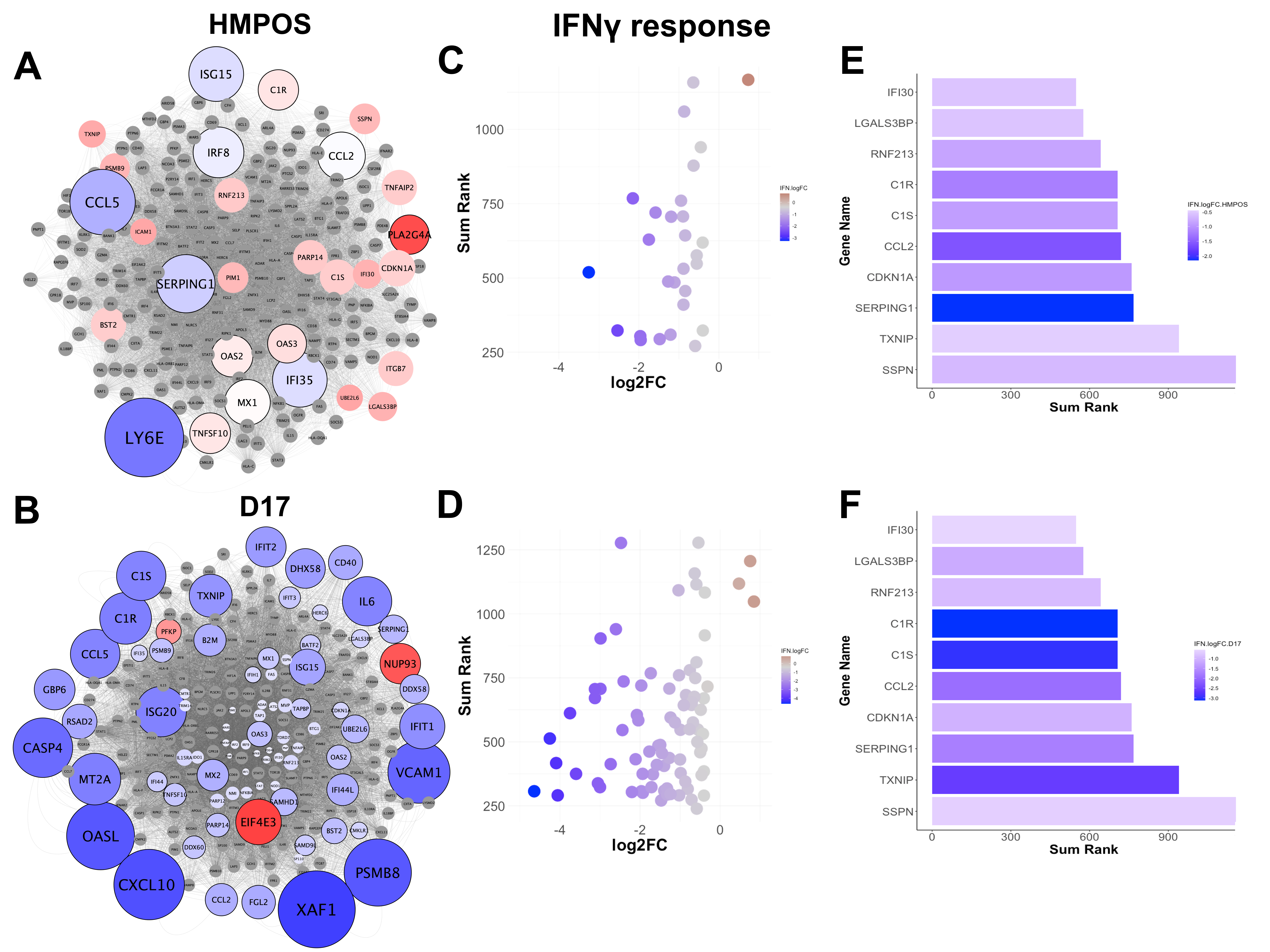

Supplement: Supplementary Figure 3 — Extravasated circulating tumor cells downregulate the interferon gamma response pathway. (A) Gene regulatory networks for the interferon gamma response pathway in HMPOS and (B) D17. Blue denotes downregulation and red denotes upregulation (FDR<0.05). Nodes are scaled proportionally to log2FC, with larger nodes depicting greater log2FC between extravasated and parental cell lines. (C) Scatter plot of sum rank of network connectivity parameters by log2FC for HMPOS and (D) D17. (E) Bar graphs of top connected genes in the pathway, colored by log2FC for HMPOS and (F) D17. [file Image_3.tif]
